# Neutrophil-lymphocyte ratio as a risk factor for osteoporotic vertebrae fractures and femoral neck fractures

**DOI:** 10.1097/MD.0000000000032701

**Published:** 2023-01-20

In the article, “Neutrophil-lymphocyte ratio as a risk factor for osteoporotic vertebrae fractures and femoral neck fractures,”^[1]^ which published in Volume 101, Issue 48 of *Medicine*, Donglai Wang was added as the final author in the author byline and the corresponding author Kepeng Li is no longer listed as the corresponding author.
